# Sandwich Enzyme-Linked Immunosorbent Assay for Detecting Sesame Seed in Foods

**DOI:** 10.1155/2015/853836

**Published:** 2015-12-09

**Authors:** Stef J. Koppelman, Gülsen Söylemez, Lynn Niemann, Ferdelie E. Gaskin, Joseph L. Baumert, Steve L. Taylor

**Affiliations:** ^1^Food Allergy Research and Resource Program, Department of Food Science and Technology, University of Nebraska, Lincoln, NE 68588-6207, USA; ^2^The Ministry of Agriculture and Rural Affairs, Directorate of Food Control and Central Food Research, 16036 Bursa, Turkey

## Abstract

Small amounts of sesame can trigger allergic reactions in sesame-allergic patients. Because sesame is a widely used food ingredient, analytical methods are needed to support quality control and food safety programs in the food industry. In this study, polyclonal antibodies against sesame seed proteins were raised, and an enzyme-linked immunosorbent assay (ELISA) was developed for the detection and quantification of sesame seed residue in food. A comparison was made between this ELISA and other assays, particularly focusing on recovery of sesame seed residue from different food matrices. The developed ELISA is sensitive with a lower limit of quantification of 0.5 ppm and shows essentially no cross-reactivity with other foods or food ingredients (92 tested). The ELISA has a good recovery for analyzing sesame-based tahini in peanut butter, outperforming one other test. In a baked bread matrix, the ELISA has a low recovery, while two other assays perform better. We conclude that a sensitive and specific ELISA can be constructed based on polyclonal antibodies, which is suitable for detection of small amounts of sesame seed relevant for highly allergic patients. Furthermore, we conclude that different food products may require different assays to ensure adequate quantification of sesame.

## 1. Introduction

Sesame (*Sesamum indicum*) is a seed crop native to Africa, the Middle East, and parts of Asia where it traditionally forms an important part of the human diet. Sesame is one of the oldest cultivated crops owing to its nutritional value and good resistance to draught. Currently, Myanmar is the largest producer of sesame seeds, followed by India and China (FAOSTAT database from the Food and Agricultural Organization of the United Nations) with an annual production of approximately 70,000 tons, compared to the US production of approximately 2,500 tons annually. Traditionally, sesame seeds are consumed as a paste (tahini and a sweetened form called halvah) that serves as ingredient for many dishes. Also, in particular in the Western countries, sesame seeds are used for toppings on bakery products like bagels, hamburger buns, and crackers. Sesame oil is also popular due to its unique flavour profile. The recent focus on health foods, vegetarian foods, and ethnic foods in the Western countries has led to an increase in the consumption of sesame seeds and sesame-derived products, with the black and white sesame being the most commonly consumed varieties. In parallel, concerns have emerged about the allergenicity of sesame seeds. Compared to allergies for milk and egg, the prevalence of sesame allergy is low and varies regionally [1]. In USA, the prevalence of sesame allergy is estimated at 0.1% based on a nationwide, cross-sectional, random telephone survey that included a total of 13,534 subjects from 5,300 households [2]. Sesame seed allergy is more common in Israel [3] where sesame seed accounts for a large part of the severe IgE-mediated reactions to food [4] and represents the third leading cause of food allergy after milk and egg. Differences between countries in the prevalence of sesame seed allergy are likely due to dietary habits. In the Middle East, sesame seeds are introduced in the diet at an early age as a good source of calories and bioavailable iron. Thus, sensitization to sesame seed and clinical sesame seed allergy before age of 2 years is documented [4].

Allergic reactions to the ingestion of sesame seed can be triggered by doses as low as 1 mg of sesame seed protein for individual cases, and the population threshold study showed that approximately 5 mg of sesame seed protein triggers a reaction in 10% of the sesame seed-allergic population [5]. Sesame seeds contain several allergenic proteins that have been identified and partially characterized [6–9]. Sesame oil can also present a hazard to sesame seed-allergic individuals as it is often not highly refined and thus contains protein residues [10].

Unintentional ingestion of sesame seed is a risk for sesame seed-allergic consumers. The presence of sesame seeds and ingredients derived from sesame seeds is not always obvious. Sesame seed flour, paste, and oil are not easily identified if products are not appropriately labelled. The seeds are small and a few seeds can easily be indistinguishable in a bakery formulation. Sesame seed residues can also occur in foods from cross-contact through use of shared processing equipment and facilities. A need exists for analytical methods that are able to sensitively and specifically detect residues of sesame seed. Immunoassays for the detection of sesame seed proteins [11, 12] and PCR methods for the detection of DNA fragments [13, 14] from sesame seed have previously been described, and commercial immunoassay kits exist for the detection and quantification of sesame seed residues. Preliminary data from our group indicated that the various matrices in which sesame seed can potentially be present complicated the quantification of sesame seed residues in food products. While some methods seem appropriate for bakery products, others may be more suitable for tahini. The aim of this study is to develop an ELISA for the detection and quantification of sesame seed residues in foods and to compare its performance with regard to sensitivity and recovery from various food matrices with two commercially available immunochemical assays for sesame seed.

## 2. Materials and Methods

### 2.1. Immunogen Preparation

Black (India black) and white (Mexico white hulled) sesame seeds were purchased from Penzeys Ltd. (Penzeys Spices, Madison, WI) and stored at room temperature until needed. Sesame seeds were washed thoroughly 1 : 8 (w/v) with distilled water 6 times and dried under a fume hood. After drying, the seeds were ground to uniform consistency using a blender (Oster Professional Products, Chicago, IL). Ground white and black sesame seeds were mixed and defatted by washing with ethyl ether (1 : 5 w/v) with continuous stirring for 5 minutes; the process was repeated 5 times. The defatted residue was then allowed to settle for 30 minutes and after that time the supernatant was discarded. At the last washing step, the mixture was collected using a Buchner funnel fitted with Whatman number 1 filter paper (Whatman International Ltd., Maidstone, England), and a final washing of the defatted sesame seeds was done twice with acetone (1 : 5 w/v). The defatted material was then dried under a fume hood at room temperature for 48 hours. This material was used for the immunization of animals. The crude protein content of the material was determined using the Kjeldahl method. Immunogen was prepared by emulsifying the dried material with adjuvant, rather than first making an extract, in order to target the largest possible diversity of epitopes including those from lipophilic proteins. Complete Freund's adjuvant (CFA, Difco Labs, Detroit, MI) was used for preparting the primary immunogen and incomplete Freund's adjuvant (IFA) (Difco Labs, Detroit, MI) was used to prepare immunogen for booster immunizations.

### 2.2. Polyclonal Antibody Production

One goat was immunized with 1.0 mg of defatted sesame seed immunogen emulsified with CFA and administrated subcutaneously (s.c.). The first booster injection, comprised of 500 *μ*g sesame seed immunogen in IFA, was administered 2 weeks after the initial immunization. Subsequent booster injections were administered with 250 *μ*g of defatted sesame seed in IFA every 3 weeks after that, alternating between s.c. and intramuscularly (i.m.). The first test bleed was taken after the second booster injection (approximately 5 weeks after the initial injection), and thereafter test bleeds were taken 10 days after each booster injection.

Three chickens were initially immunized with defatted sesame seed immunogen in CFA, followed by booster injections at 2-week intervals using 200 *μ*g in IFA (s.c. and i.m., alternatively) basically according to Schade et al. [15]. Eight weeks after the initial immunization, this group was rested for approximately 6 weeks, and then booster injections were changed to 3-week intervals due to an observed decrease in antibody production.

Titer development was monitored using a noncompetitive ELISA, basically as described by Hefle et al. [16]. In short, microtiter plates (MaxiSorp, Nagle Nunc International, Roskilde, Denmark) were coated with 1 *μ*g sesame seed protein/mL of coating buffer (0.015 M Na_2_CO_3_, 0.035 M NaHCO_3_, and 0.02% NaN_3_, pH 9.6) and incubated overnight at 4°C. After blocking with 350 *μ*L of PBS containing 0.1% gelatin (porcine, 300 bloom, Sigma Chemical Co., St. Louis, MO), sera from goat or chicken egg yolks were incubated in various dilutions. Plates were washed with PBS containing 0.05% Tween 20, and bound goat IgG and egg IgY were detected and quantified using commercial anti-immunoglobulin-alkaline phosphatase conjugates (anti-goat IgG [Pierce Chemical Co., Rockford, IL]; anti-chicken IgY [Promega Co., Madison, WI]), respectively, following the manufacturer's instructions. Bleeds from the goat with titers above 10,000 were pooled. Yolks with titers >3,000 were pooled.

### 2.3. Antibody Isolation

Polyclonal IgG antibodies were partially purified from the sera by precipitation in 50 and 35% ammonium sulfate [16]. The partially purified IgG was briefly dialyzed against deionized water and then extensively dialyzed against 0.01 M phosphate-buffered saline (PBS) (0.002 M NaH_2_PO_4_, 0.008 M Na_2_HPO_4_, 0.85% NaCl, and 0.02% NaN_3_, pH 7.4) as described by Hefle et al. [16]. This IgG fraction was used as the coating antibody for ELISA development. Immune eggs were collected and stored at 4°C until needed. The IgY fraction was purified using a commercial EGGstract IgY purification system (Promega Co., Madison, WI). Immunoglobulin Y preparations were stored at −20°C until needed. This IgY fraction was used as detection antibody in the ELISA development. The protein content of the IgG (48.5 mg/mL) and IgY (7.7 mg/mL) fractions was determined by the Lowry method [17].

### 2.4. Gel Electrophoresis and Immunoblotting

The reactivity of the polyclonal chicken and goat antibodies was characterized by means of sodium dodecyl sulfate-polyacrylamide gel electrophoresis (SDS-PAGE) and immunoblotting. Samples of white sesame and black sesame extract (10 *μ*g/well) were separated under reducing conditions using the Laemmli [18] method with minor modifications. Electrophoresis was performed using a Bio-Rad Mini Protean II (Bio-Rad Laboratories) with Ready Gel precast gels (15% Tri-HCl; Bio-Rad Laboratories). Proteins were located on the gels by staining with brilliant blue G-colloidal stain and then scanned with the Kodak Gel Logic 440 imaging system (Kodak Scientific Imaging Systems, New Haven, CT, USA). Proteins from unstained SDS-PAGE gels were transferred to polyvinyl difluoride (PVDF) membranes (Immobilon-P PVDF membrane, 0.45 *μ*m, Millipore Corporation, Billerica, MA, USA) as described by Towbin et al. [19]. The membranes were cut such that part of the lanes could be stained for protein using India ink. The remaining part of the membranes was used for immunoblotting and was blocked with 0.2% BSA in PBS, pH 7.4. Chicken egg yolk (IgY) and goat (IgG) antibodies to sesame seed were tested in 5,000- and 10,000-fold dilutions as primary antibodies using PBS-T (0.05% Tween 20) containing 0.2% BSA RIA grade (USB Corporation, Cleveland, OH, USA). Then the membrane was incubated with either commercial anti-chicken IgY or anti-goat IgG peroxidase conjugates (Promega Co., Madison, WI, and Pierce Chemical Co., Rockford, IL, resp.). The membranes were washed three times with PBS containing 0.05% Tween 20 (PBS-T) and then treated for at least 15 minutes with DAB (3′3-diaminobenzidine) substrate solution (Pierce Biotechnology, Inc.) prepared according to the manufacturer's recommendation. After washing with PBS-T, the membranes were air-dried overnight at room temperature. Digital images of the stained, dried PDVF membranes from both protein staining and immunoblotting experiments were then captured using a Kodak Gel Logic 440 imaging system and analyzed with the Kodak Gel Logic ID v.3.6.5 software (Kodak Scientific Imaging Systems).

### 2.5. Sandwich ELISA

The general approach for the development of the sandwich ELISA was as follows. Optimal concentrations of coating antibody and detection antibody were determined by varying the concentration of the coating antibody in one dimension of microtiter plates and the concentration of the detecting antibody in the other dimension, using various antigen concentrations and the relevant controls. Antibody concentrations were chosen such that the sensitivity of the ELISA was sufficient while the nonspecific binding (background) was still low. This led to the following ELISA protocol. Microtiter plates (MaxiSorp, Nagle Nunc International, Roskilde, Denmark) were coated with 100 *μ*L of goat anti-sesame seed antibody at 1 *μ*g/mL in coating buffer (0.015 M Na_2_CO_3_, 0.035 M NaHCO_3_, and 0.02% NaN_3_, pH 9.6) and incubated overnight at 4°C. All subsequent incubations were carried out at 37°C for 1 h (except incubation for color development with substrate). Plates were washed with PBS containing 0.05% Tween 20 and 0.02% NaN_3_ (PBS-T) after each incubation step using a programmable automatic plate washer (Dynatec Laboratories Inc., Chantilly, VA). Plates were blocked with 350 *μ*L of PBS containing 0.1% gelatin (porcine, 300 bloom, Sigma Chemical Co., St. Louis, MO) per well for one hour. Samples and standards were diluted in incubation buffer (PBS buffer containing 0.1% bovine serum albumin, RIA grade, Sigma Chemical Co., St. Louis, MO) and applied to wells in triplicate on each plate with a one-hour incubation. After washing three times, 100 *μ*L of anti-sesame seed chicken egg yolk IgY antibody at 1.75 *μ*g/mL in incubation buffer was then added and incubated for one hour, followed by washing four times. Then, 100 *μ*L of commercial goat anti-chicken IgY-alkaline phosphatase conjugate (Promega, Madison, WI) was added to each well diluted 2,000-fold in incubation buffer, followed by a one-hour incubation. After washing four times, 100 *μ*L of* p-*nitrophenyl phosphate (SigmaFast, Sigma, St. Louis, MO) was added and incubated for 30 min at room temperature for color development. Color development was stopped by addition of 100 *μ*L of 0.1 N NaOH. The absorbance was measured at 410 nm using a MR5000 plate reader (Dynatech Laboratories Inc., Chantilly, VA).

### 2.6. Standard Curve for Sandwich ELISA

Standard curves were constructed with extracts of bread mix spiked with known amounts of sesame in the form of sesame seed flour. Thereto, 454 grams of dry bread mix (Hodgson Mills, lot number 10 07 09 2) was mixed with required amount of sesame seed flour to achieve 1,000 ppm in the final recipe. This amount will result in a concentration of 0.01% or 100 ppm once the other ingredients are added (total weight of 742 g). Another 454 grams of dry bread mix served as negative control. To 454 grams of dry bread mix, 30 grams of vegetable oil, 6 grams of fresh yeast, and 252 grams of water were added, resulting in a total weight of 742 grams. Extracts were made of the 1,000 ppm spiked bread mix and 0 ppm spiked bread mix (as described below) and these extracts were mixed in different ratios to obtain the dilutions required for the standard curve. Standard curves were generated using Prism graphics software (GraphPad Prism, Inc., San Diego, CA).

### 2.7. Comparison with Commercial Sesame Seed Assays

Commercially available ELISA kits for sesame seed were obtained from Tepnel Biokits (Deeside, United Kingdom) and ELISA Systems (Windsor, Australia). Assays were provided as ready to use kits, with all reagents, diluents, and standards included and were performed according to the manufacturer's instructions. The Tepnel Biokits assay had a claimed range of detection of 6 to 100 ppm, the ELISA Systems assay of 0.5 to 5 ppm. Extracts were prepared according to the instructions of the kit manufacturers.

### 2.8. Food Samples

All food samples were purchased from local retailers in Lincoln, Nebraska. Ninety-seven foods or food ingredients were evaluated for cross-reactivity and matrix effects in the ELISA. All samples were ground to uniform consistency using an Oster blender (Oster Professional Products, Chicago, IL) or a mortar and pestle depending on the texture of the samples.

### 2.9. Naturally Incurred Food: Bread Spiked with Sesame Seed Flour

Bread mix spiked with 100 ppm sesame seed (as sesame flour) and negative control bread mix were prepared as described for the preparation of the standard curve of the sandwich ELISA. The spiked bread mix and the negative control bread mix were mixed in different ratios such that concentrations of 100 ppm, 50 ppm, 10 ppm, 5 ppm, 1 ppm, and 0 ppm were reached in the final bread. From these mixes, bread was baked using an automatic bread baker (default settings). After cooling to room temperature, samples were taken from different locations from the bread.

### 2.10. Naturally Incurred Food: Peanut Butter Spiked with Tahini

Tahini and peanut butter were obtained from a local supermarket. A spiked standard of 10,000 ppm sesame seed in peanut butter was prepared by mixing tahini and peanut butter in a 1/100 (W/W) ratio. The mix was blended extensively using a kitchen blender. The 10,000 ppm product was used to spike peanut butter in the following concentrations: 100 ppm, 50 ppm, 10 ppm, 5 ppm, 1 ppm, and 0 ppm. All samples were blended extensively before analysis.

### 2.11. Preparation of Extracts for ELISA

Samples seeds were ground with a coffee grinder before extracting. Preliminary data indicated that less rigorous methods of grinding gave relatively low recoveries when intact sesame seeds were present (not shown). Ground samples were extracted 1 : 10 (w/v) in 0.01 M PBS for 2 h at room temperature with gentle shaking using a Labquake shaker (Barnstead/Thermoline, Dubuque, IA). Extracts were clarified by centrifugation at 3,200 ×g at 10°C in a Sorvall Centrifuge (Kendro Laboratory Products, Newtown, CT). Fresh extracts were used and stored at 4°C for up to 5 days.

Extracts for cross-reactivity testing were tested in undiluted form, 10-fold diluted from, and 100-fold diluted form, and results were calculated as ppm sesame seed equivalence using 1,000,000 ppm reactivity for sesame seed by definition.

## 3. Results and Discussion

### 3.1. Characterization of Antibodies

**Figure 1:**
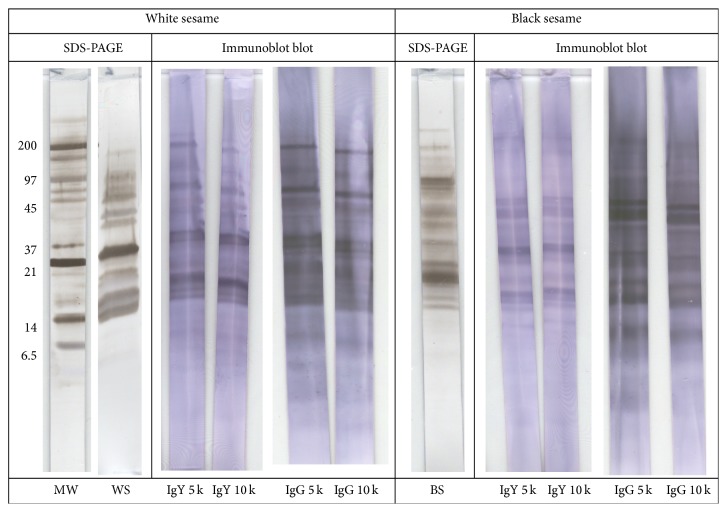
SDS-PAGE and immunoblot of sesame. MW: molecular weight markers (indicated left in kDa), WS: white sesame extract, and BS: black sesame extract. Blots developed with egg yolk (IgY) or sheep (IgG) antibodies, diluted 5,000-fold (5 k) or 10,000-fold (10 k).

Crude extracts of white and black sesame seed were used for developing polyclonal antibodies for a sandwich ELISA to detect and quantify sesame seed residues in foods. The advantage of polyclonal antibodies compared to monoclonal antibodies is that polyclonal antibodies are more robust in detecting antigens in food products that have been exposed to food processing. A monoclonal antibody may become less reactive if its epitope is affected by food processing. For polyclonal antibodies, more epitopes play a role and overall reactivity will only partly be affected when a certain epitope is affected by food processing. Furthermore, because we choose to work with whole extracts rather than purified or isolated proteins, the number of epitopes is even larger, diminishing the chance of losing reactivity in the ELISA when the reactivity of one of the epitopes is affected by food processing. The goat polyclonal IgG antibody that was used for coating recognizes a broad range of sesame seed proteins, both for black and white sesame seed. The polyclonal IgY antibody that was used as the detection antibody was raised in chickens and obtained from egg yolk. This polyclonal antibody also recognized a broad range of proteins both in black and white sesame seeds (Figure 1). Both polyclonal antibodies show good reactivity for all major protein bands present in both extracts. Proteins in black sesame seed extract, at approximately 20 and 60 kDa, were comparably somewhat less reactive. Several sesame seed allergens have been identified in the past decades. An early study reported that proteins in the range of 14–25 kDa were important IgE-binding proteins for a group of 12 French patients [6]. In a study with 20 sesame seed-allergic patients from USA, Beyer et al. [7] found IgE binding towards sesame seed proteins in the range from 7 to 78 kDa. The proteins with the lower molecular weight were assigned as Ses i 1 and Ses i 2 and appear to be 2S albumins, known as potent allergens from other seeds, nuts, and legumes as well. Beyer et al. [7] found that 75% of their patients had IgE-reactivity to the 45 kDa 7S albumin, a protein with a high sequence homology with the important peanut allergen, Ara h 1. This allergen was assigned Ses i 3. Leduc et al. [8] identified two other potentially important sesame seed allergens as oleosins in the 15–17 kDa range in a French population of 32 sesame seed-allergic patients; these two oleosins were designated as Ses i 4 and Ses i 5. Work on serology of an Italian patient population (*n* = 18) indicated an important role for the 11S globulin, Ses i 6 and Ses i 7, in particular its basic subunit of approximately 25 kDa [9]. Although we did not identify the proteins and allergens that our polyclonal antibodies were reactive to, we see good reactivity in the low molecular weight region typical for 2S albumins and oleosins, probably reflecting reactivity to Ses i 1, Ses i 2, Ses i 4, Ses i 5, and/or the basic subunit of Ses i 6 and Ses i 7. Furthermore, we see clear reactivity of both polyclonal antibodies at the 45 kDa region, probably corresponding to Ses i 3. Because sesame seed is used as a food ingredient as the intact seed, as flour, or as a paste made from the whole seed, it is unlikely that individual allergens become separated from each other during food processing. Therefore, if a set of antibodies recognizes several proteins or allergens, they are potentially suitable for use in development of an ELISA to detect sesame seed residues in a range of food products.

### 3.2. Sensitivity of the ELISA

**Figure 2:**
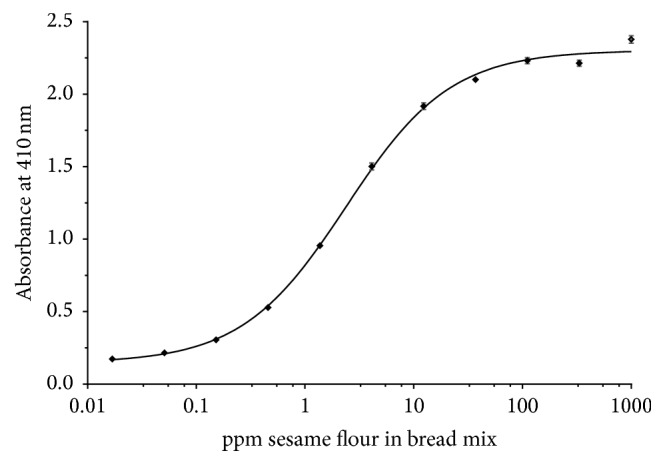
Calibration curve of sesame ELISA. Data points are mean of nine independent experiments; error bars represent the standard deviation.

With the polyclonal antibodies raised in goat and chicken, a sandwich ELISA was developed using the goat antibody as coat and the chicken egg yolk IgY antibody as the detector. The sandwich ELISA was tested for sensitivity by measuring a range of known concentrations of sesame seed flour extract spiked into a bread mix. In Figure 2, a typical example of the average of 9 independents assays is shown. The sensitive range is from 0.15 to 37 ppm of sesame seed in bread mix. The lowest tested standard was 0.02 ppm; however the response of this sample was close to the background signal. Samples of 0.5 ppm consistently gave absorbances that were well above the background signal and we therefore used 0.5 ppm as lower limit of quantification (LLOQ). Interpolation between 0.5 and 0.02 ppm can be done, but the calculated results may be imprecise.

Limited information exists regarding the minimum dose of sesame seed proteins needed to elicit an allergic reaction. Double-blind, placebo controlled food challenge (DBPCFC) threshold studies with large numbers of patients are available for some allergenic foods such as peanut [20–22]. The population threshold for sesame seeds can also be estimated but less precisely due to the lack of data from sesame seed-allergic individuals [5, 22]. The Allergen Bureau of Australia and New Zealand has established a Reference Dose for sesame seed of 0.2 mg sesame seed protein as a part of their VITAL program based upon statistical dose distribution modelling of available published data on individual sesame seed thresholds [22]. As with peanut [20, 22], considerable variability seems to exist regarding individual threshold doses among sesame seed-allergic patients. There are a few studies that report levels of sesame seeds that caused reactions. One study based on 9 sesame-allergic patients from France showed that allergic reactions were triggered with 100 mg sesame seed flour [23] while other patients also from France reacted at substantially higher dosages of 10 grams [6]. The lowest reported threshold for sesame protein was 1 mg [5], corresponding to 2 mg of seed flour. In a population of 35 individuals, 5 mg of sesame protein (corresponding to 10 mg sesame flour) triggered a reaction in 10% of the sesame-allergic patients [5]. Case reports also appear to indicate that small amounts of sesame seed [24] or sesame-based tahini [25] can elicit severe allergic reactions, but the exact amount ingested was not known. In one of the cases, the threshold dose for the individual was determined afterwards and appeared to be 2 grams [24]. The statistical models used in the VITAL program are inherently conservative and no evidence exists of sesame seed-allergic patients reacting to 0.2 mg of sesame seed protein (0.67 mg sesame seed flour based upon 30% protein in sesame flour or 1.2 mg sesame seed based on 17% protein in sesame seed) [5, 22]. Using the conservative lower threshold of 1 mg sesame seed and assuming an intake of 100 grams per portion, a relevant concentration of sesame residue that may trigger an allergic reaction is 10 ppm. The LLOQ of our assay (0.5 ppm) is well below this value and therefore suitable to detect sesame seed residue present in foods at clinically relevant concentrations.

Other assays to detect sesame seed residues in food products have been described previously based on either immunochemical methodology or on DNA-amplification methodology. The sensitivity of the immunochemical methods varied from 0.6 ppm sesame protein (sandwich ELISA [12]) to 5 ppm sesame protein (inhibition ELISA [11]). The sensitivity of the DNA-amplification method was in one report 50 ppm [13] and 5 ppm in another report [14]. At least one of these methods by Schöringhumer et al. [13] may not detect clinically relevant levels of sesame seed residues based upon the VITAL Reference Dose [22]. Furthermore, the DNA-amplification methods detect DNA rather than the allergenic proteinaceous part of sesame seeds. Results obtained with DNA-amplification methods should therefore be interpreted with more care especially for processed food products containing sesame seed-based ingredients where the allergenic protein part is separated from the DNA/RNA fraction.

### 3.3. Specificity of the ELISA

The specificity of the sandwich ELISA was investigated by testing extracts of 92 food ingredients in serial dilutions.  Table 1 shows the cross-reactivity of these food ingredients, expressed as ppm equivalence of sesame seed flour, in which the reactivity of sesame itself is defined as 1,000,000 ppm sesame seed flour. The majority of the evaluated samples were negative at all tested dilutions. About a quarter of the samples were somewhat responsive in their undiluted form, and only a few were still reactive at a 10-fold dilution. For the 100-fold dilutions, only kidney beans and allspice were just above two times the background, resulting in a cross-reactivity of 3.3 and 2.0 ppm sesame seed flour, respectively. Compared to sesame seed, this is still very low, more than 100,000-fold less than sesame seed. While the specificity of the DNA-based methods for allergen detection is often very high as was the case for sesame [13, 14], immunoassays based on polyclonal antibodies are sometimes not very specific. The indirect competitive ELISA using a single polyclonal antibody was developed by Husain et al. [11] which showed substantial cross-reactivity of 0.7% (or 7,000 ppm sesame seed protein) for one of the 13 tested food ingredients. The combination of different, independently raised polyclonal antibodies into a sandwich ELISA as was done in our study can at least partially overcome this issue [12]. The high specificity of our method allows applying the method for a wide variety of food products. Interestingly, sesame oil did not react in our ELISA, presumably because the oil was sufficiently refined and did not contain detectable amounts of (sesame) protein. Similar observations of absence of allergen in oils were also made for peanut oil [26] and mustard oil [27].

### 3.4. Comparing Different Immunoassays for Sesame Seed: Recovery from Different Matrices

Food processing conditions, in particular heat treatment like baking, often result in decreased recovery of allergens when assayed with immunoassays. Also, food products that have a high-fat content are known to be difficult matrices for detection of allergen residues [28]. For sesame seed, the recovery from whole grain bread was described to be lower than expected [11]. We compared our currently developed assay with two commercially available assays for detection of sesame seed residue with regard to recovery from difficult food matrices. Two model food products were prepared: bread baked from flour spiked with sesame seed flour and peanut butter spiked with tahini. For the commercial assays, the extraction procedure recommended by the kit manufacturer was used.

Model breads were baked with known amounts of sesame seed flour spiked into the bread mix before preparing the dough. After baking, samples were taken from different locations in the bread (middle part, lighter crust end, and darker crust end).  Table 2 shows the recovery from the different samples for the three tested assays. Some test results were below the LLOQ of the respective assays. These values were estimated by analyzing diluted standards and interpolation between the lowest tested standard and negative control. This approach is not truly quantitative, but because it gives an indication of the recovery of sesame seed in food products spiked with a low level of sesame seed, these values were included in Table 2. There are clear differences between the assays. The current method and the kit from ELISA Systems show poor recoveries. The mean recoveries for the current method and the ELISA Systems kit were 6.5% and 13%, respectively. Neither of these two assays could detect the lowest spike concentration of 1 ppm sesame flour. The Tepnel-Neogen kit showed a better recovery with a mean of 39%. It was expected that the middle part of the bread would show the highest recovery because this was less intensively exposed to heat, while the darker crust end would show the lowest recovery. However, there was no correlation between exposure to heat and recovery (Table 2).

Our sesame seed ELISA was also compared to the two commercial ELISA kits after spiking peanut butter with known amounts of tahini (Table 3). As explained for Table 2, diluted standards were again used to obtain information on the recovery of sesame seed residue in food products spiked with a low level of sesame seed paste. Again, this is not a true quantitation but data are included in Table 3 for informational purposes. For the tahini model food product, the current assay showed the best recovery (mean: 83%), while the Tepnel-Neogen assay led to an overestimation of the sesame content (mean recovery: 239%). The assay from ELISA Systems had a mean recovery of 6% and could not detect the lowest spike concentration of 1 ppm seed paste.

Recovery of food allergens from food products for detection and quantitation has been topic of investigation for quite some time. The majority of the published work is on peanut detection. Recovery from chocolate products and cookies has been tested for different (commercially available) assays in ring trials. One particular study that included 5 different assays for peanut residues and 30 European laboratories found recoveries ranging from 44 to 191% for low ppm spikes [29]. For sesame seed assays, there are no such comprehensive ring trials. Of the two reported immunoassays to detect sesame seed residues, the recoveries ranged from 70 to 160% [11] and 42 to 145% [12]. No other assays were used in these studies, and only bakery products were tested, limiting the general applicability of their data to a broader range of food products.

Many factors play a role in detectability of allergens by immunoassays. Limiting factors may be epitope recognition, solubility/extractability, grinding efficiency, and in-homogeneity of the food products [28]. Because it is not known which antigens were used to prepare the antibodies applied in the commercial ELISAs, differences in antibody specificity may also affect the recovery. With regard to epitope recognition, we use two independent sources of polyclonal antibody, recognizing multiple proteins in sesame seed (Figure 1), and probably multiple epitopes per protein as well. This reduces the risk of poor recognition when a specific part of a protein is affected or damaged during food processing. Solubility and extractability depend on the matrix and the extraction buffer. Apparently, our method works well for tahini, although this is a high-fat matrix. Grinding and inhomogeneity of the samples may be an issue for bakery products where whole sesame seeds are often used as topping. Preliminary data from our lab showed that different grinding procedures result in different extraction yields and recoveries in our ELISA; this is a topic of further investigations. The poor recovery from bakery products may also be the result of limited solubility/extractability and this may be the reason that our assay shows a poor recovery when testing bakery products (Table 2). Baking is known to decrease the solubility and extractability of proteins, leading to low recoveries in ELISAs [30, 31]. Some manufacturers of commercial immunoassays to detect allergens in food products add an extraction additive to improve extractability from difficult matrices like chocolate or baked products. Optimizing grinding and extraction procedure may be useful to further develop our ELISA. The two commercially available assays we evaluated in this study also show remarkable differences in recovery, which were in some cases relatively low. It may turn out that certain food products should preferably be analyzed with the one assay, while other types of food product require another assay for optimal analysis.

## 4. Concluding Remarks

A set of polyclonal antibodies for the detection of sesame seed protein was developed. The sandwich ELISA constructed with these antibodies was sensitive and specific for the detection of sesame seed residues. However, the recovery from baked food products was low. Other assays appear to have better recoveries for certain food products. The current data show that different assays may be needed to measure sesame seed residues reliably in different food products and that great care should be taken when assessing the level of sesame seed residues in food products.

## Figures and Tables

**Table 1:** Cross-reactivity of food ingredients in the sesame sandwich ELISA.

Ingredient description	Cross-reactivity (ppm)
Almonds (raw)	<1
Anise seed	1.01
Apple (fresh)	1.12
Apple (dried)	<1
Apricot (dried)	<1
Banana (fresh)	<1
Banana (dried)	<1
Bell peppers (green, fresh)	<1
Bell peppers (red, fresh)	<1
Bell peppers (red, roasted)	<1
Brazil nuts	<1
Cacao	<1
Canola oil	<1
Caraway seed	1.22
Cashew	<1
Celery seed	1.55
Cherries (sweet, pitted)	1.26
Cherries (tart red, pitted)	1.02
Cheese (Parmesan)	<1
Cheese (Romano)	<1
Chick peas (fresh)	<1
Chick peas (dried)	<1
Chocolate (dark)	<1
Chocolate (milk)	<1
Cinnamon	1.89
Cloves	<1
Coconut	<1
Corn meal	<1
Corn starch	<1
Corn syrup (dark)	<1
Corn syrup (light)	<1
Cumin	1.59
Curry powder	1.49
Dates (dried, pitted)	1.18
Fennel seed	1.63
Garlic (fresh)	<1
Garlic powder	1.23
Hazelnut (roasted)	<1
Honey	<1
Kidney beans	3.34
Kiwi (fresh)	<1
Lemon (fresh)	<1
Macadamias (roasted)	<1
Mace	1.84
Mango (dried, sweetened)	<1
Marjoram	<1
Milk (1% low fat milk)	<1
Molasses (unsulphured)	1.18
Mushroom (portobello)	<1
Mushroom (shiitake)	<1
Mustards seed (yellow)	<1
Nutmeg	1.83
Oat (whole grain)	<1
Onion (fresh)	<1
Onion (dried powder)	<1
Oregano	<1
Paprika	1.67
Parsley flakes (dried)	<1
Peaches (fresh)	1.01
Peanut (roasted)	<1
Peas (green, dried)	<1
Pecan (fresh)	1.03
Pecan (roasted)	<1
Pepper (black)	1.45
Pine nuts	<1
Pistachios (dry roasted)	<1
Raisins	<1
Raspberries	1.30
Rice (dried)	<1
Rye (flour, whole grain)	<1
Salt	<1
Semolina	<1
Sesame oil (extra virgin)	<1
Soy flour	<1
Soy milk	<1
Soy nuts (roasted, salted)	<1
Soy oil	<1
Strawberry (fresh)	<1
Sugar (white)	<1
Sugar (light brown)	<1
Sunflower seeds (salted)	<1
Thyme (dried)	<1
Tomato paste	<1
Tomato puree	<1
Vanilla extract	<1
Walnuts (black)	<1
Wheat flour	<1
Yeast (active, dried)	<1

Mix: bread flour	<1
Mix: cake flour	<1
Mix: spices (unspecified)	2.05

**Table 2:** Recovery of sesame form bread spiked with sesame flour using different ELISA methods.

Bread sample	Spike level	Current method^1^		Neogen-Tepnel^2^		ELISA Systems^3^	
		Sesame (ppm)	Recovery (%)	Sesame (ppm)	Recovery (%)	Sesame (ppm)	Recovery (%)
Middle part	0	—	n.a.	—	n.a.	—	n.a.
	1	—	n.a.	0.34	34	0.16	16
	5	0.43	9	1.45	29	0.85	17
	10	0.61	6	4.56	46	1.15	12
	50	2.28	5	24.3	49	6.5	13
	100	6.53	7	21.8	22	14.4	14

Lighter crust end	0	BLQ	n.a.	—	n.a.	—	n.a.
	1	0.18	18	0.95	95	0.18	18
	5	0.49	10	2.6	52	0.63	13
	10	0.49	5	4.3	43	1.18	12
	50	2.97	6	17.5	35	5.73	11
	100	7.29	7	42.3	42	13.3	13

Darker crust end	0	—	n.a.	—	n.a.	—	n.a.
	1	—	n.a.	0.41	41	0.14	14
	5	0.17	3	0.61	12	0.6	12
	10	0.31	3	2.8	28	1.09	11
	50	1.55	3	16.6	33	5.62	11
	100	2.05	2	16.6	17	12	12

1: defined LLOQ is 0.5 ppm. Values between 0.1 and 0.5 ppm may be therefore imprecise.

2: LLOQ given by manufacturer is 6 ppm. Values below 6 ppm may be therefore imprecise.

3: LLOQ given by manufacturer is 0.5 ppm. Values below 0.5 ppm may be therefore imprecise.

n.a.: not applicable.

**Table 3:** Recovery of sesame form peanut butter spiked with tahini using different ELISA methods.

Spike level	Current method		Neogen-Tepnel^1^		ELISA Systems^2^	
	Sesame (ppm)	Recovery (%)	Sesame (ppm)	Recovery (%)	Sesame (ppm)	Recovery (%)
0	—	n.a.	—	n.a.	—	n.a.
1	0.71	71	2.53	253	0.09	9
5	3.84	77	12.4	248	0.32	6
10	7.51	75	24.1	241	0.53	5
50	43.2	87	119	238	2.09	4
100	104	104	214	214	4.11	4

1: LLOQ given by manufacturer is 6 ppm. Values below 6 ppm may be therefore imprecise.

2: LLOQ given by manufacturer is 0.5 ppm. Values below 0.5 ppm may be therefore imprecise.

n.a.: not applicable.
